# Controlling the Mdm2-Mdmx-p53 Circuit

**DOI:** 10.3390/ph3051576

**Published:** 2010-05-18

**Authors:** David L. Waning, Jason A. Lehman, Christopher N. Batuello, Lindsey D. Mayo

**Affiliations:** 1 Herman B Wells Center for Pediatric Research, 980 West Walnut, Walther Hall R3-C548, Indianapolis, IN 46202, USA; 2 Department of Biochemistry and Molecular Biology, Indiana University School of Medicine, 635 Barnhill Drive, MS 4053, Indianapolis, IN 46202, USA

**Keywords:** Mdm2, Mdmx, p53, phosphorylation, kinase inhibitor

## Abstract

The p53 tumor suppressor is a key protein in maintaining the integrity of the genome by inducing either cell cycle arrest or apoptosis following cellular stress signals. Two human family members, Mdm2 and Mdmx, are primarily responsible for inactivating p53 transcription and targeting p53 protein for ubiquitin-mediated degradation. In response to genotoxic stress, post-translational modifications to p53, Mdm2 and Mdmx stabilize and activate p53. The role that phosphorylation of these molecules plays in the cellular response to genotoxic agents has been extensively studied with respect to cancer biology. In this review, we discuss the main phosphorylation events of p53, Mdm2 and Mdmx in response to DNA damage that are important for p53 stability and activity. In tumors that harbor wild-type p53, reactivation of p53 by modulating both Mdm2 and Mdmx signaling is well suited as a therapeutic strategy. However, the rationale for development of kinase inhibitors that target the Mdm2-Mdmx-p53 axis must be carefully considered since modulation of certain kinase signaling pathways has the potential to destabilize and inactivate p53.

## 1. Introduction

The tumor suppressor protein p53, plays key role in maintaining the integrity of the genome by inducing either cell cycle arrest or apoptosis following cellular stress signals [1]. p53 is activated in response to DNA damage, ribosomal stress, oncogene activation, hypoxia, nutrient deprivation and telomere erosion [2]. Greater than half of all human cancers harbor mutations in p53 that renders it inactive while almost all malignancies target p53 for functional inactivation. Loss of p53 function confers a growth advantage for cancer cells. Therefore, it is not surprising that p53 is a highly regulated protein. 

Mdm2 and Mdmx are oncoproteins that have essential yet non-redundant roles as the major negative regulators of p53. Mdm2 is an E3 ubiquitin ligase that targets p53 for proteasome-dependent degradation [3,4,5]. Mdm2 and Mdmx bind to the amino terminal transactivation domain of p53 to block transcriptional activity [6,7,8,9]. Robust activation of p53 is predicated on the removal of Mdm2 and Mdmx. As such, Mdm2 and Mdmx have been extensively studied in terms of their response to DNA damage and regulation of p53 protein levels and activity.

p53, Mdm2 and Mdmx are targets for varied post-translational modifications following genotoxic stress. To protect p53, several signaling pathways induced by genotoxic stress alter the ability of Mdm2 and Mdmx to neutralize p53. For Mdm2 this is largely through inhibition of Mdm2-mediated ubiquitination of p53 whereas for Mdmx this is mainly by inhibiting the transactivation domain of p53. In response to DNA damage, Mdm2 and Mdmx post-translational modifications are mainly phosphorylation through multiple kinases [10,11], while direct p53 regulation occurs through phosphorylation as well as sumoylation, neddylation and acetylation [12]. 

**Figure 1:**
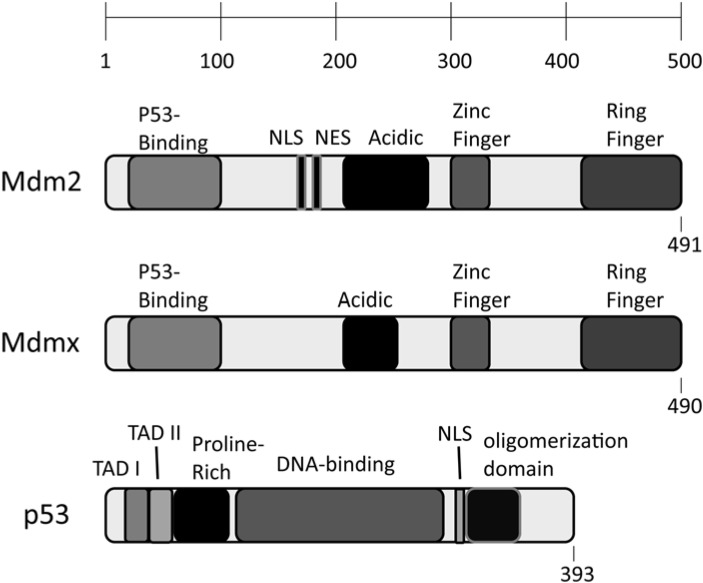
Schematic representation of the domains of Mdm2, Mdmx and p53.

p53 is comprised of an amino terminal set of transactivation domains (TAD I at residues 20–40 and TAD II at residues 40–60) important for selective gene targeting. A proline rich domain at residues 60–90, is important for apoptotic activity. The central DNA binding domain of p53 spans residues 100–300 followed by a nuclear localization signal domain from residues 315–325. Active p53 requires oligomerization, which is controlled by the oligomerization domain between residues 300–350 [13] (Figure 1). Mdm2 and Mdmx share considerable domain structure with Mdmx having a slightly shorter acidic domain and lacking nuclear localization and nuclear export signal domains. Mdm2 and Mdmx both harbor an amino terminal p53 interacting domain (residues 25–110). This domain is critical for inhibiting the transcriptional activity of p53. Mdm2 (but not Mdmx) has nuclear localization and nuclear export sequences between residues 175 and 195. The central acidic domain of Mdm2 and Mdmx (residues 200–280), is important for target selection and ubiquitination. Mdm2 and Mdmx also both have a zinc finger domain near residues 300–330. Finally, the RING domain of Mdm2 (residues 435–490) is important for Mdm2 homo-dimerization, Mdm2-Mdmx hetero-dimerization and ubiquitin ligase activity (Figure 1). The RING domain of Mdmx is important for oligomerization with minimal ubiquitin ligase activity [14]. 

In this review, we will focus on the phosphorylation events that are involved in the regulation of the p53 pathway. Most studies of the post-translational modifications in the Mdm2-Mdmx-p53 axis are in response to genotoxic agents. Clinical development of small molecule inhibitors against kinases involved in these signaling pathways has mainly been aimed at cancer therapeutics. Thus, our review will focus on kinases that regulate the Mdm2-Mdmx-p53 axis.

## 2. Regulating p53 through Phosphorylation

Signaling pathways emanating from DNA damage regulate the Mdm2-Mdmx-p53 axis [15]. Of significant importance for the Mdm2-Mdmx-p53 axis are ATM (ataxia telangiectasia mutated) kinase, ATR (ataxia telangiectasia and Rad3 related) kinase and DNA-PK (DNA-dependent protein kinase) pathways. ATM and DNA-PK pathways are predominantly activated by DNA double strand breaks whereas ATR is activated mainly by lesions in the DNA induced by UV or DNA cross-links that lead to stalled replication forks [16,17,18]. Once activated, ATM, ATR and DNA-PK all phosphorylate components of the DNA damage response and lead to modifications of p53 and Mdm2 and to some degree at least, Mdmx [17]. These modifications ultimately stabilize p53 and lead to its transcriptional activation [19].

### 2.1. Phosphorylation of p53 after DNA damage

Phosphorylation plays a role in the stabilization of p53 following DNA damage [12,20]. p53 is modified by a range of kinases some of which overlap the kinases that target Mdm2 and Mdmx (see below). Phosphorylation of p53 in response to DNA damage occurs mainly in the amino terminal transactivation domain [12] (Table 1). Phosphorylation of p53 usually drives p53 transcriptional activation since these modifications stabilize p53. In human cells, ionizing radiation (IR) and ultraviolet light (UV) lead to extensive phosphorylation in the transactivation domain of p53 (serines 6, 9, 15, 20, 33, 37, 46, and threonines 18 and 81) [21]. IR and UV also induce phosphorylation at the carboxy terminus of p53 (serines 315 and 392) [22,23]. Adding to the potential for complexity in regulation, threonines 55, 150, 155 and serine 149 in the central region of p53 [24,25,26] and serines 376 and 378 [27,28] of p53 are phosphorylated under homeostatic conditions and may become hypo-phosphorylated following genotoxic stress. Interestingly, several kinases are capable of phosphorylating the majority of target sites of p53. This redundancy indicates the importance of p53 in tumor suppression and allows a mechanism for fine-tuning the control of p53 responses by various signaling pathway inputs [12].

Phosphorylation of serine residues near the p53 amino terminus (especially serines 15 and 20) is important for stabilization of p53 by decreasing association with Mdm2 and possibly Mdmx [29,30]. However, it does not appear that these residues are solely responsible for stabilization since mouse knock-in mutations of the corresponding murine sites (serines 18 and 23) show limited affect in certain tissues. This indicates that phosphorylation of these sites may not be a universal requirement for stabilization of p53 [31]. ATM is the primary kinase for p53 serine 15 leading to enhanced transcriptional activation. The importance of this modification has been shown by *in vitro* methods [32] and through expression of phospho-mimetic substitutions [33,34]. ATM also activates the checkpoint kinase Chk2 [35]. Chk2 phosphorylates p53 at serine 20 and interferes with the p53-Mdm2 interaction serving to stabilize p53 (see above). While ATM and Chk2 seem to be most important following IR, ATR is required for efficient response to UV damage in human cells through phosphorylation of p53 at serines 15 and 37 [36]. 

DNA damage also leads to phosphorylation of p53 by additional kinases (Table 1). Notable are, casein kinase 1 delta (CK1) that phosphorylates p53 at serine 9 and threonine 18 in a cascade of events that depends on the upstream phosphorylation of p53 at serines 6 and 15 [37,38,39,40]. The activity of CK1 serves to stabilize p53 by blocking interaction with Mdm2. Mass spectrometric and antisense experiments have shown that c-Jun N-terminal kinase (JNK) phosphorylates p53 at threonine 81 in response to DNA damage [41]. Homeodomain-interacting protein kinase 2 (HIPK2) has been shown to phosphorylate p53 at serine 46 both *in vitro* and in response to DNA damage *in vivo* [42,43]. These and other studies have shown that differences in the phosphorylation pattern of p53 exist in response to various sources of DNA damage. These complex and interconnected signaling mechanisms give some indication to the versatility and adaptability of the p53 response.

### 2.2. Phosphorylation of Mdm2 after DNA damage

Phosphorylation of Mdm2 is localized to four main regions that are induced either by mitogenic signals or DNA damage (Table 2). Mitogenic signals lead to phosphorylation of a group of four serine residues near the nuclear localization and nuclear export sequences (serines 157, 166, 186 and 188). These sites will not be considered further in this article but have been reviewed elsewhere [11]. In response to DNA damage, Mdm2 is modified at the amino terminus, within the central acidic domain and within a disperse group near the carboxy terminal RING domain. Mdm2 serine 17 near the amino terminus is phosphorylated by DNA-PK *in vitro* [44]. More recent biochemical studies have shown that this site is responsible for dictating the dynamic equilibrium of Mdm2-p53 interactions [45]. Under homeostatic conditions, a large group of serine residues (including: serines 240, 242, 246, 253, 256, 260, 262 and 269) in the acidic domain are phosphorylated. This region becomes hypo-phosphorylated under stress conditions [11,46,47]. The acidic domain is important for target recruitment and ubiquitination [48]. DNA damage also leads to phosphorylation of a more disperse group of serine and tyrosine residues mainly residing near the RING domain (tyrosine 394, serines 386, 395, 407, 425, 428 and threonine 419) with an additional site adjacent to the acidic domain (tyrosine 276). 

**Table 1:** DNA damage induced p53 phosphorylation.

Site	Kinase	Activation	Outcome
Serine 15	ATM	DNA damage	apoptosis
Serine 15, 37	ATR	gamma, UV	apoptosis
Serine 315	CDK	UV	p53 transcription
Serine 20	Chk1/Chk2	IR, UV	inhibition of p53-Mdm2 complex
Serine 149, Threonine 150, 155	CSN kinase complex	homeostatic	p53 degradation
Serine 15, 37	DNA-PK	DNA damage	inhibition of p53-Mdm2 complex
Serine 15, Threonine 55	ERK	UV, DNA damage	apoptosis
Serine 392	CK2	UV	p53 transactivation
Serine 46	HIPK2	UV	apoptosis, acetylation of p53
Serine 20, Threonine 81	JNK	UV, DNA damage	p53 stabilization and apoptosis
Serine 20	MAPKAPK2	UV	apoptosis
Serine 15, 33, 46, 392	p38 kinase	UV, DNA damage	p53 stabilization and apoptosis
Serine 376 and 378	PKC	homeostatic	p53 degradation
Threonine 55	TAF1	homeostatic	p53 degradation

ATM, ataxia telangiectasia mutated; ATR, ataxia telangiectasia and Rad3 related; CDK, cyclin-dependent kinase; Chk1, checkpoint kinase; CSN kinase, COP9 signalosome kinase; DNA-PK, DNA-dependent protein kinase; ERK, extracellular signal-regulated kinase; CK2, casein kinase II, HIPK2, homeodomain-interacting protein kinase 2; JNK, c-Jun NH_2_-terminal kinase; MAPKAPK2, mitogen-activated protein kinase-activated protein kinase2; PKC, protein kinase C; TAF1, component of TFIID.

DNA damage activates cell cycle checkpoints that lead to the robust activation of ATM and ATR kinase pathways. ATM is activated by DNA double strand breaks while ATR is activated by stalled replication forks [49]. Direct phosphorylation of Mdm2 at serine 395 by ATM blocks nuclear export of p53 and leads to stabilization of p53 protein [32,50]. ATM phosphorylation of Mdm2 at serine 386, 395, 425 and 428, and at threonine 419 cooperatively lead to stabilization of p53 by preventing poly-ubiquitination, a consequence of preventing Mdm2 RING domain homo-dimerization [51]. ATR phosphorylates Mdm2 at serine 407 in response to specific types of DNA damage blocking nuclear export of p53 [52]. ATM also activates the downstream c-Abl kinase through direct phosphorylation in response to DNA damage [53,54,55]. c-Abl phosphorylates Mdm2 at tyrosines 276 and 394 [56,57]. Phosphorylation of Mdm2 tyrosine 276 leads to increased levels of nucleolar Mdm2 and increases binding of Mdm2 to its negative regulator, ARF. Thus ARF protects p53 through re-localization of Mdm2 [56]. Phosphorylation of Mdm2 tyrosine 394 stabilizes p53 and inhibits the negative regulation of Mdm2 on p53 transcriptional and apoptotic activities [57]. An additional c-Abl target site at Mdm2 tyrosine 405 has been identified but a physiological role has not been determined [56]. These events support a multi-factorial model of Mdm2 regulation based on varied signaling events.

### 2.3. Phosphorylation of Mdmx after DNA damage

As with Mdm2, Mdmx is also phosphorylated at multiple sites in response to DNA damage (Table 2). ATM phosphorylation of Mdmx at serine 403 leads to rapid degradation of Mdmx alleviating repression of p53 activity [58,59]. ATM-dependent Chk2 phosphorylation of Mdmx at serine 367 increases binding to the adapter protein 14-3-3, which has been suggested to compete with the de-ubiquitinating enzyme HAUSP leading to destabilization of Mdmx [59,60,61]. In addition, Mdmx serines 161, 342, 365 and 391 are also phosphorylated in response to DNA damage but their relative contribution to Mdmx regulation is not known [11]. Wang YV and co-authors have generated a mouse that harbors a series of three conserved serine-to-alanine mutations in Mdmx, sites that become phosphorylated in response to DNA damage. The authors report that these mice lack robust Mdmx degradation in response to DNA damage and that this compromises p53 activity [62]. This result highlights the *in vivo* importance of these modifications in control of the cellular response to stress. Recently it has been shown that c-Abl phosphorylates Mdmx at tyrosines 55 and 99. Phosphorylation of Mdmx at tyrosine 99 inhibits Mdmx-p53 complex formation, which frees p53 to activate gene expression [63]. Also, casein kinase 1 alpha (CK1α) has been shown to phosphorylate Mdmx at serine 289 in the acidic domain. Knockdown of CK1α or ionizing radiation leads to the activation of p53 and apoptosis but the molecular mechanism remains to be determined [64]. Thus varied responses to DNA damage have the potential for multiple levels of control with regard to the Mdmx response.

**Table 2:** DNA damage induced Mdm2 and Mdmx phosphorylation.

Site	Kinase	Activation	Outcome
**Mdm2**			
Serine 17	DNA-PK		block Mdm2-p53 binding
Tyrosine 276, 394, 405	c-Abl	MTC, DXR, IR	stabilize p53
Serines 240, 242, 246, 253, 256, 260, 262 and 269		homeostatic	E3 ligase target substrate recruitment modulation, stabilize p53 after IR
Serine 386, 395, 425, 428 and Threonine 419	ATM	IR, UV, NCS	stabilize and activate p53
Serine 407	ATR	CPT	down-regulate nuclear export of p53
**Mdmx**			
Serine 403	ATM	NCS, IR, ETO	destabilize Mdmx
Serine 342, 367	Chk2	NCS, IR	destabilize Mdmx
Tyrosine 55, 99	c-Abl		block Mdmx-p53 binding
Serine 289	CK1a	IR	activation of p53

ATM, ataxia telangiectasia mutated; ATR, ataxia telangiectasia and Rad3 related; c-Abl, cellular Abelson kinase; Chk2, checkpoint kinase; CK1a, casein kinase 1 alpha; DNA-PK, DNA-dependent protein kinase; NCS, neocarzinostatin; DXR, doxorubicin, MTC, mitomycin C; CPT, camptothecin, ETO, etoposide.

## 3. Kinase Inhibitors of the Mdm2-Mdmx-p53 Axis

The search for therapeutic kinase inhibitors has accelerated in the past decade with the majority of research and development efforts aimed at the treatment of cancer. The reasons for the current interest in kinases as therapeutic targets are varied. There are greater than 500 kinases encoded by the human genome. Since signal transduction pathways predominantly involve phosphotransfer, many kinases are involved in processes that lead to tumor formation. Cell cycle and growth pathways are hyperactive in cancer and the normal control mechanisms that prevent kinase activation are often lost. Cells can also lose their responsiveness to growth factors due to aberrant kinase activity in mitogenic signaling cascades. Thus, selective pharmacological compounds aimed at kinase activity have been successfully developed and approved for use in humans. Kinase inhibitors are usually well tolerated in normal cells allowing for selective treatment of tumor cells as the tumor cells often become addicted to signaling pathways provided by kinases [65]. 

The multiple kinase signaling cascades that affect p53 are cumulatively important for full engagement of the tumor suppressive activities of p53. These include both the direct phosphorylation of p53 as well as modifications to p53’s negative regulators, Mdm2 and Mdmx. The focus of this review is to identify the kinase modification events that target the Mdm2-Mdmx-p53 axis in response to DNA damage. Table 3 lists important kinase inhibitors that target signaling events of Mdm2, Mdmx and p53.

**Table 3:** Kinase inhibitors that directly and indirectly target Mdm2, Mdmx and p53.

Kinase	Inhibitor
c-Abl	imatinib^a^ [66], dasatinib^a,b^ [67,68], nilotinib^a,b^ [69], ON012380^c^ [70]
ATM	KU-55933^a^ [71], KU-60019^a^ [72], CP466722 [73]
CDK	SNS-032 [74], AT7519^a^ [75]
Chk1/Chk2	AZD7762^a^ [76]
CSN	curcumin
DNA-PK	morphlins [77], NU-7441^a^ [78], NU-7422^a^ [79], LY294002^a^ [80].
ERK	lapatinib^a^ [81]
JNK	SP600125^a^ [82]
MAPKAP2	pyrazinoindolone [83], subersic acid [84], makassaric acid [84]
PKC	ingenol 3-angelate^c^ [85,86], midostaurin^a^ [87], safingol [88]
TAF1	apigenin^a^ [89]

^a^ Type I inhibitors; ^b^ Type II inhibitors; ^c^ allosteric inhibitors; ^d^ irreversible covalent inhibitors.

### 3.1. Classes of kinase inhibitors

Protein kinases are able to catalyze the transfer of the terminal phosphate of ATP to a target substrate. Protein kinases either target serine and threonine residues or tyrosine residues around some amino acid sequence specificity or structural specificity motif. ATP binding is typically in a deep pocket of the kinase active site. The majority of kinase inhibitors target the ATP binding site for competitive binding [90]. Four different classes of kinase inhibitors have been identified. Type I kinase inhibitors represent the largest class of kinase inhibitors and are competitive inhibitors of the kinase active conformation. Type II kinase inhibitors recognize the inactive conformation of the kinase typically through a hydrophobic patch near the ATP binding site that is only exposed in the inactive conformation. In addition to compounds that target the ATP binding site, a third type, the allosteric kinase inhibitors have been developed that modulate kinase activity. These compounds exhibit the highest degree of selectivity since their binding sites are independent of the well-conserved kinase active site. This class of compounds also includes inhibitors that bind accessory molecules that are required for kinase activity. The fourth type of inhibitor is covalent inhibitors that form irreversible crosslinks to the kinase active site rendering it inactive [65]. In addition to the current compounds in development or trials, a large group of analogues that have modifications to the basic chemistry of the original lead compound are being designed to provide enhanced selectivity or lower toxicity. 

### 3.2. Kinase inhibitors that target the Mdm2-Mdmx-p53 axis

Over the past decade pharmaceutical and academic researchers have begun to understand and target kinase-signaling pathways that are involved in cancer development and metastasis. Much work has led to the appreciation that targeting kinases in cancer will likely require some rationalization of drug selection based on individualized patient criteria. Patient specific differences arise from the type of tumor and the tumor microenvironment. Understanding the tumor in the context of its kinase-dependent growth characteristics will aid selection of treatment regimens. Understanding the kinase signaling pathways involved in loss of growth control affords the clinician some therapeutic rationale for treatment. 

Understanding the interplay of Mdm2 and Mdmx with p53 in tumor cells would aid drug selection. Dysregulation of p53 function plays a critical role in tumor development by side stepping p53-dependent responses. Inactivation of p53 in tumors is achieved through two main mechanisms. First, inactivation of p53 function by direct mutation of p53 and second, by disrupting signaling pathways that lead to p53 activity. For tumors harboring wild-type p53, re-activating p53 in established tumor cells represents an effective intervention scheme [91,92]. In more than half of tumors with non-functional p53, the p53 protein is wild type. In these cases, affecting p53 activity directly or through modulation of Mdm2 and/or Mdmx to re-activate p53 activity would likely lead to therapeutically favorable responses. Of particular interest are therapies that might exert less selective pressure on cells while exerting their effects on multiple targets (e.g. Mdm2 and Mdmx). There is little doubt that drugs that activate a functional p53 pathway would have wide applications in the treatment of cancer. 

Modulating Mdm2 and Mdmx levels has profound effects on p53 activity. Low expression levels of Mdm2 or Mdmx is lethal whereas an excess of either can be oncogenic [93]. Many human tumors express high levels of either Mdm2 or Mdmx [94]. In fact, a modest two-fold increase in Mdm2 protein is sufficient for tumorigenesis [95,96]. Additionally, a single nucleotide polymorphism (SNP) in the *Mdm2* promoter that increases Mdm2 mRNA and proteins levels on the order of two- to four-fold is a strongly correlated with poor prognosis [97]. Further, deletion of one allele of Mdm2 or Mdmx in mice suppresses B-cell lymphoma development induced by the oncogene c-Myc [98]. These data taken with the fact that signal transduction pathways: (1) are responsible for the nuclear import and export of Mdm2, (2) alter Mdm2 ubiquitin ligase activity, (3) affect Mdm2 binding partners and (4) affect Mdm2 regulatory functions suggests that selectively targeting the kinases that modulate Mdm2 and Mdmx activity would protect and activate p53. Thus providing novel therapeutic targets.

The classic example of a rationally designed kinase inhibitor is the Abelson tyrosine kinase (Abl) inhibitor imatinib [66]. The use of imatinib to treat chronic myelogenous leukemia (CML) presents a case study of the need for a careful understanding of the disease mechanism and drug action in predicting drug applicability for other indications. Imatinib inhibits the Abl kinase activity of the constitutively active mutant BCR-Abl fusion kinase protein by blocking ATP binding. In addition, imatinib is minimally toxic to non-disease cells [99]. BCR-Abl is the result of a gene fusion between the break-point cluster region (BCR) gene and c-Abl kinase (translocation t(9;22) or Philadelphia chromosome). BCR-Abl is present in 95% of patients diagnosed with CML [100]. BCR-Abl functions as an oncogene by dysregulating intracellular signaling leading to aberrant proliferation and resistance to apoptosis. The clinical outcome of the BCR-Abl fusion gene product is an abundance of myeloid progenitor and differentiated cells. At the time of diagnosis, CML patients typically have peripheral blood counts nearly 20-fold higher than normal [101]. Blood cells harboring the BCR-Abl fusion gene product initially maintain their normal activity but eventually lose their ability to differentiate leading to blast crisis. Imatinib is much less effective after blast crisis presumably due to the presence of multiple “hits” to the cell. Imatinib provides positive cellular response in 65–90% of patients with CML and up to 80–90% response when patients are in early chronic phase [102,103,104]. Imatinib is generally well tolerated with few side effects compared to standard cytotoxic chemotherapy. Low peripheral blood counts are a common side effect with imatinib treatment while non-hematologic reactions are minor [101]. Imatinib is a success story of rationalized drug design but also illustrates a need for multifaceted approaches in cancer treatment [66].

The initial excitement of imatinib’s success was dampened by the early identification of resistance mutations mainly in the BCR-Abl kinase domain [105]. Resistance to imatinib in CML is usually by the reactivation of BCR-Abl signal transduction. Imatinib resistance in CML develops quickly, and some argue inevitably, since the selective pressures on cells treated with single target therapies is high. Since cells exposed to single target therapies only need to overcome a single source of inhibition, a further point mutation is often sufficient to develop resistance. And due to the rapid proliferation of cancer cells, the rise of resistance mutations often occurs in a clinical setting.

Imatinib has also been used on a limited basis for treatment of other tumors with mixed success. Imatinib exhibited a lack of response in at least one study with metastatic Leydig cell tumor [106]. Further, in a mouse model of mammary adenocarcinoma cells (4TI, p53 null), imatinib treatment lead to accelerated tumor growth [107]. These results suggest that the reported *in vitro* and animal model findings for imatinib may not be directly applicable for additional indications [108,109]. These disparate results suggest that a more complicated signaling cascade is at play in various tumor models. Since CML is typified by hyperactive Abl kinase activity, imatinib is useful in reducing the level of Abl kinase activity in the cell to a more normal physiological level. However, pressures for tumor growth eventually overtake the action of the drug and resistance mutants develop. The action of imatinib in cells that have normal Abl signaling would produce a whole different range of signaling events that may or may not be advantageous as cancer therapeutics. In this context, treatment of tumors harboring wild-type p53 with imatinib would not likely provide benefit since p53 levels would be negatively impacted through inhibition of Abl kinase activity. Additionally, blocking Abl phosphorylation of Mdmx would cause the formation of Mdmx-p53 complexes, rendering p53 transcriptionally inactive [63].

## 4. Conclusions

The application of kinase inhibitors for the treatment of cancer is currently a major focus in drug development. These compounds have relatively few side effects and show very good initial efficacy. However, development of compounds with further specificity is a challenge and the rise of resistance mutations limits the clinical impact of any single target compound. Rational use of several compounds that selectively target multiple kinases in a single cascade may provide a mechanism to lessen drug resistance in the clinic. In the case of p53, this could theoretically be accomplished by blocking a kinase-signaling cascade common to both Mdm2 and Mdmx. However, a thorough understanding of the signaling events impacted by a drug is needed to ensure that beneficial kinase signaling is not blocked. A balanced approach of targeting kinases known to negatively regulate p53 activity while maintaining those that activate p53 presents a logical means of target selection.

Drug development, especially early on in the development cycle, requires a better mechanistic understanding and predictive capacity to mitigate the possibility of drug resistance. Also, more predictive tumor models are required since some of the animal models are not fully and faithfully recapitulated in human tumors. Finally, a more sophisticated modeling of inhibitors in various tumors with associated tumor microenvironment constraints would be useful to elucidate the role of a specific kinase inhibitor in the context of the vastly interconnected signaling circuits present in cells.
